# Personalization in Modern Radiation Oncology: Methods, Results and Pitfalls. Personalized Interventions and Breast Cancer

**DOI:** 10.3389/fonc.2021.616042

**Published:** 2021-03-18

**Authors:** Cynthia Aristei, Elisabetta Perrucci, Emanuele Alì, Fabio Marazzi, Valeria Masiello, Simonetta Saldi, Gianluca Ingrosso

**Affiliations:** ^1^Radiation Oncology Section, University of Perugia and Perugia General Hospital, Perugia, Italy; ^2^Radiation Oncology Section, Perugia General Hospital, Perugia, Italy; ^3^Radiation Oncology Section, University of Perugia, Perugia, Italy; ^4^Radiation Oncology Department, Fondazione Policlinico A. Gemelli IRCCS, Rome, Italy

**Keywords:** breast cancer, personalized medicine, precision medicine, radiation oncology, biomarkers, molecular subtypes, gene profiles, genetic assay

## Abstract

Breast cancer, the most frequent malignancy in women worldwide, is a heterogeneous group of diseases, characterized by distinct molecular aberrations. In precision medicine, radiation oncology for breast cancer aims at tailoring treatment according to tumor biology and each patient’s clinical features and genetics. Although systemic therapies are personalized according to molecular sub-type [i.e. endocrine therapy for receptor-positive disease and anti-human epidermal growth factor receptor 2 (HER2) therapy for HER2-positive disease] and multi-gene assays, personalized radiation therapy has yet to be adopted in the clinical setting. Currently, attempts are being made to identify prognostic and/or predictive factors, biomarkers, signatures that could lead to personalized treatment in order to select appropriate patients who might, or might not, benefit from radiation therapy or whose radiation therapy might be escalated or de-escalated in dosages and volumes. This overview focuses on what has been achieved to date in personalized post-operative radiation therapy and individual patient radiosensitivity assessments by means of tumor sub-types and genetics.

## Introduction

Breast cancer is the most frequent malignancy in women worldwide. On the basis of clinical level 1 evidence, current international guidelines recommend adjuvant systemic and radiation treatments, as well as the radiation therapy (RT) volumes to be irradiated, dose delivery and fractionation schedules after breast conserving surgery (BCS) and mastectomy. Personalized approaches are needed as, rather than one disease with varying histological features and clinical behavior, breast cancer is a heterogeneous group of diseases, characterized by distinct molecular aberrations (1). Personalized medicine, which accurately assesses risk factors for tumor recurrence or progression at all care stages from diagnosis to surgery, therapy and follow-up, already dictates choice of systemic therapy for breast cancer patients. Endocrine therapy (ET) is prescribed for hormonal receptor-positive disease and anti-human epidermal growth factor receptor 2 (HER2) therapy for HER2-positive disease. In early-stage disease, multi-gene assays (i.e. Oncotype DX^®^ Breast Recurrence Score (RS) (Genomic Health Inc., Redwood City, CA, USA), MammaPrint^®^ (Agendia BV, Amsterdam, Netherlands), Prosigna^®^ (PAM50; NanoString Technologies Inc, Seattle WA, USA), EndoPredict^®^ (Myriad Genetics Inc, Salt Lake City, UT, USA), Breast Cancer Index^®^ (BCI) (NeoGenomics Laboratories, Fort Myers, FL, USA) (2–4) may be offered as prognostic tools to estimate the risk of distant recurrence. Their results may lead to tailored adjuvant systemic therapies i.e. prolonged ET, ET alone or chemotherapy before ET (2, 4–7). Finally, studies are investigating the potentialities of immunotherapy in the adjuvant setting in triple negative (TN) disease (8).

Unlike systemic therapy, fully personalized RT has yet to be adopted in the clinical setting (9) as standard clinical-pathological parameters like patient’s age, tumor size, nodal involvement, margin width, hormone receptor status, tumor grade, lymphovascular invasion still drive adjuvant RT. Current treatment planning includes contouring patient-specific target volumes and organs at risk of toxicity while beam angles, shapes, and energies are individually defined so that personal dose-volume histograms are selected to ensure an optimal treatment choice and delivery for each patient. Advanced RT techniques such as IMRT, VMAT, or tomotherapy result in better dose homogeneity within the target volume and allow for a reduction of higher doses to the organs at risk (e.g., heart, lungs) (10). Despite these advantages, modulated RT techniques are still not considered standard of care and, consequently, are reserved for selected cases, such as when regional nodes need to be irradiated, breasts are voluminous and when patients present an unfavorable anatomy. Research is advancing into proton irradiation for selected patients as these particles deliver the dose to a specified depth, thus lowering the risk of cardiac and pulmonary toxicity (11–21). Even though there has been a clear increase in proton facilities in recent years, availability remains scarce, evidence supporting its clinical use is limited, and costs are high (22, 23). Another fast-growing research area in radiation oncology is radiomics which uses data-characterization algorithms to extract features from radiological images, detect patterns, and uncover cancer characteristics as images contain much more information than perceived by the imaging interpreter or the clinician. In the field of breast cancer, interest in radiomics has grown significantly in recent years, as clinicians attempt to elucidate intrinsic biological factors and discover how they shape therapeutic responses. Linking radiomics information to disease stratification, prognosis, and therapeutic response could provide valuable information for personalized therapy (24–26) but, unfortunately, to date no study has linked radiomics information with RT outcomes.

**Figure 1:**
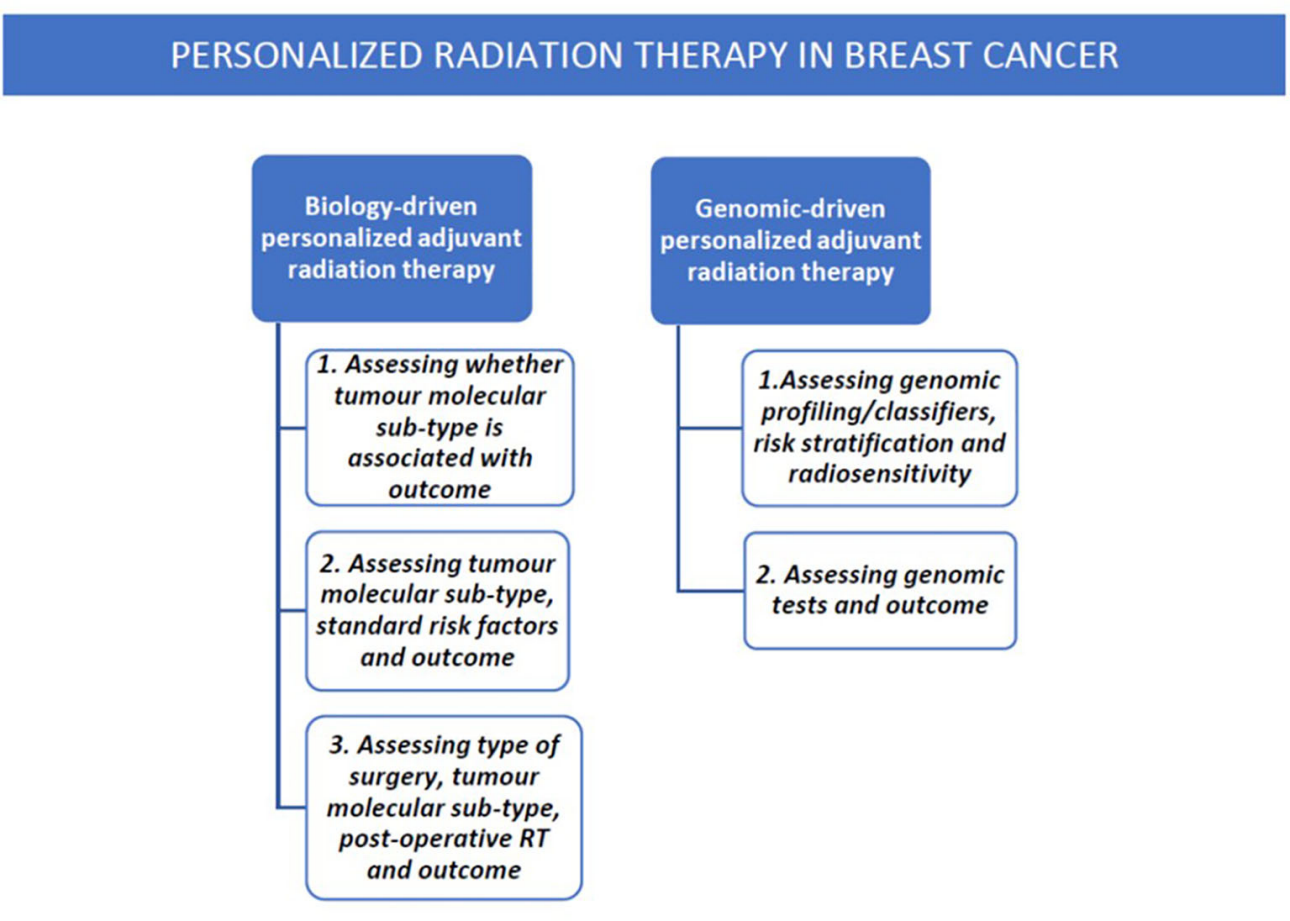
Flow chart of the present overview’s topics.

Since not all patients with breast cancer benefit from RT, and its benefit is not equal across risk groups, a current challenge is to identify suitable candidates as no specific biomarkers are available to guide decision-making. In order to improve cure and survival rates and/or reduce toxicity, attention is focused at present on identifying prognostic and/or predictive factors, biomarkers, signatures so as to aid decision-making in whether or not to administer RT and escalate or de-escalate dosages and volumes. Research is currently investigating protein or phenotypic markers, molecular sub-types, new classifiers, and genomic signatures in attempts to decipher the tumor’s genetic fingerprint or surrogate sub-type and associated risk of local or loco-regional relapse (LR, LLR) which may determine post-operative RT. This overview hopes to throw some light on the topic by reviewing studies on radiosensitivity as assessed by tumor sub-types and genetics (**Figure 1**).

## Sources of Information

From May 2020 to September 2020, Pubmed and the Cochrane library were searched for relevant literature.

## Biology-Driven Personalized Adjuvant Radiation Therapy

As outcomes vary greatly after BCS and mastectomy, clinical studies have been conducted since the beginning of this century to establish the impact of molecular sub-types on LR, LRR, distant metastases (DM), and overall survival (OS) and their links with well-known risk factors for relapse, type of surgery, and RT.

### Assessing Whether Tumor Molecular Sub-Type Is Associated With Outcome

One of the earliest studies enrolled 482 patients (24% TN) treated with BCS and RT from 1980 to 2003; 75% were node negative and outcomes were analyzed at a median follow-up of 7.9 years. Compared with all other molecular sub-types, TN did not correlate with local control; TN patients had significantly worse distant metastasis-free survival and cause-specific survival (27). Another study of 1,601 patients [180 TN (11.2%) *vs* all others] confirmed that no significant difference emerged in local recurrence rates. TN was associated with a shorter median time to local recurrence (2.8 *vs* 4.2 years) and was linked to a significantly worse probability of being distant recurrence-free and breast cancer specific survival free (28).

Other retrospective studies showed that sub-type was a prognostic factor for outcome. In a series of 793 patients who were treated with BCS followed by RT from 1998 to 2001, all other sub-types were compared with Luminal A (595/793; 75%). Multivariate analysis showed that the adjusted hazard ratio of LR was 7.1 for basal type tumors and 9.2 for HER2-positive. In univariate analysis the adjusted hazard ratio for distant metastases was 3.9 for Luminal B, 4.6 for Basal Subtype and 5.3 for HER2-positive. However, after adjusting for tumor grade and size, number of positive nodes and use of systemic therapy, only Luminal B and the basal groups showed a significantly greater risk of distant metastases (29).

A meta-analysis of 22 studies with 15,312 patients who were treated with BCS or mastectomy ± post-operative RT showed that TN tumors were associated with a higher risk of LRR and DM than all other sub-types pooled together. In separate analyses, TN was linked to a higher risk of LRR and DM than the luminal subtypes but to a lower risk than the HER2 sub-type even though OS was the same (30). Another meta-analysis of 15 studies in which 21,645 patients had been treated with BCS (88.3% also received post-operative RT), confirmed the TN sub-type had the highest recurrence risk of all (31). **Table 1** (32–42) reports results from other studies that were not analyzed in these two meta-analyses.

**Table 1:** Studies assessing whether tumor molecular sub-type is associated with outcome which were not included in the metanalyses.

Author	N° patients	Surgery/RT	Follow-up	Outcomes and Results
Billar et al. (32)	1,061	BCS/Mastectomy ± RT	31 months	**LRR** $\begin{array}{l} \text{TN}\;\;\;\;\;\;\;\;5.7\%\\ \text{HER}2+2.9\%\\ \text{ER}+\;\;\;\;\;1.0\% \end{array}\}(p=0.001)$
Panoff et al. (33)	582	Mastectomy + RT	44.7 months	**5-y LRR** TN *vs* other combinations 11.8 *vs* 3.9% (p < 0.001)
van der Hage et al. (34)	549 <40 y 341 <40 y and N0	BCS/Mastectomy	11 years	**OS (549 pts)** Basal *vs* Luminal A HR 0.50 (95% CI 0.29–0.86) Basal *vs* HER2 HR 0.42 (95% CI 0.17–1.04) Basal *vs* Luminal B HR 0.92 (95% CI 0.56–1.48) **OS (341 pts)** Basal *vs* Luminal A HR 0.22 (95% CI 0.08–0.60) Basal *vs* HER2 HR 0.25 (95% CI 0.03–1.85) Basal *vs* Luminal B HR 0.87 (95% CI 0.48–1.59)
Wang et al. (35)	2,118	BCS/Mastectomy ± RT	67.9 months	**OS** $\begin{array}{l} \text{Luminal}\;\;\text{A}\;94.2\%\\ \text{Luminal}\;\;\text{B}\;92.6\%\\ \text{HER}2+88.7\%\\ \text{Basal}-\text{like}\;87.9\% \end{array}\}(p=0.001)$ **RFS** $\begin{array}{l} \text{Luminal}\;\;\text{A}\;87.3\%\\ \text{Luminal}\;\;\text{B}\;84.3\%\\ \text{HER}2+80.9\%\\ \text{Basal}-\text{like}\;\;79.1\% \end{array}\}(p<0.001)$
Dominici et al. (36)	819	Mastectomy + RT	58 months	**5-y LRR** $\begin{array}{l} \text{Hormonal}\;\text{Receptor}+/\text{HER}2-\;\;1\%\\ \text{Hormonal}\;\text{Receptor}+/\text{HER}2+\;6.5\%\\ \text{Hormonal}\;\text{Receptor}-/\text{HER}2+\;\;2\%\\ \text{Hormonal}\;\text{Receptor}-/\text{HER}2-\;10.9\% \end{array}\}(p<0.01)$
Tseng et al. (37)	5,673	Mastectomy + RT	50.1 months	**5-y cumulative LRR** $\begin{array}{l} \text{Luminal}\;\text{A}\;\;\;\;\;\;\;\;\;\;\;\;\;\;0.99\%\\ \text{Luminal}\;\;B\;\;\;\;\;\;\;\;\;\;2.20\%\\ \text{HER}2\;\text{without}\;\text{TR}\;\;\;\;3.60\%\\ \text{HER}2\;\text{with}\;\text{TR}\;\;\;\;0.26\%\\ \text{TN}\;\;\;\;\;\;\;\;\;\;\;\;\;5.25\% \end{array}\}(p<0.001)$
Truong et al. (38)	1,994	Mastectomy	4.3 years	**5-y LRR-free survival** $\begin{array}{l} \text{Luminal}\;\text{A}\;\;\;\;\;\;\;\;\;\;1.8\%\\ \text{Luminal}\;\;B\;\;\;\;\;\;\;\;\;\;\;1.8\%3.1\%\\ \text{Luminal}\;\text{HER}2\;\;\;\;\;1.7\%\\ \text{HER}2+\;\;\;\;\;\;\;\;\;\;\;\;\;\;\;1.9\%\\ \text{TN}\;\;\;\;\;\;\;\;\;\;\;\;\;\;\;1.9\% \end{array}\}(p=0.81)$ **5-y DR** $\begin{array}{l} \text{Luminal}\;\text{A}\;\;\;\;\;\;\;\;\;\;1.8\%\\ \text{Luminal}\;\text{B}\;\;\;\;\;\;\;\;\;\;5.0\%\\ \text{Luminal}\;\text{HER}2\;\;\;\;\;2.4\%\\ \text{HER}2+\;\;\;\;\;\;\;\;\;\;\;\;\;\;\;1.1\%\\ \text{TN}\;\;\;\;\;\;\;\;\;\;\;\;\;\;\;\;\;\;\;\;\;\;\;\;\;\;\;\;\;\;\;\;\;\;\;\;\;\;\;\;\;\;\;\;\;9.6\% \end{array}\}(p<0.001)$
Gangi et al. (39)	1,851	BCS ± RT	60 months	**5-y LRR** TN *vs* luminal A HR 1.4 (95% CI, 0.6–3.3) TN *vs* luminal B HR 1.6 (95% CI, 0.5–5.2) TN *vs* HER2 HR 1.1 (95% CI, 0.2–5.2)
Liu et al. (40)	501	BCS ± RT	10 years	**10-y IBR** $\begin{array}{l} \text{Luminal}\;\text{A}\;\;\;\;\;25.2\%\\ \text{Luminal}\;\text{B}\;\;\;\;10.5\%\\ \text{other}\;\;\;\;\;\;\;\;\;\;\;\;\;\;\;\;\;\;\;\;\;\;\;\;21.3\% \end{array}\}(p<0.001)$
Bergen et al. (41)	571 ≥65 y	NA	38 months	**DRR** $\begin{array}{l} \text{HER}2+36.1\%\\ \text{TN}\;\;\;\;\;\;\;\;\;25.4\%\\ \text{Luminal}\;14.5\% \end{array}\}(p<0.001)$
Braunstein et al. (42)	2,233	BCS ± RT	106 months	**LR** Luminal A vs Luminal-B (HR 2.64, p = 0.001) Luminal A *vs* Luminal HER2 (HR 0.93, p = 0.90) Luminal A *vs* HER2+ (HR 5.42, p < 0.001) Luminal A *vs* TN (HR 4.33, p < 0.001)

BCS, breast conserving surgery; RT, radiotherapy; LRR, loco-regional relapse; TN, triple negative; OS, overall survival; RFS: relapse-free survival; TR, trastuzumab; IBR, ipsilateral breast relapse; DRR, distant recurrence rate; LR, local relapse.

In summary, even though TN and HER2-positive tumors were reported to have the worst prognosis and Luminal A tumors the best, while Luminal B tumors were variable, intrinsic study limitations need to be kept in mind when considering the links between tumor sub-type and prognosis as several methodological flaws could have impacted on the results. All studies were retrospective, subgroup definitions were not always the same, negative estrogen receptor (ER) and progesterone receptor (PR) status was not standardized (<10 or <1%), no guidelines were available to test for HER2-positive disease, HER2-positive status at immunohistochemistry was considered negative when not investigated by fluorescence *in situ* hybridization (FISH). Luminal B were usually ER-positive, PR-positive, and HER2-positive, Ki-67 was rarely considered and G3 was sometimes used as its surrogate. Finally, some studies were conducted before trastuzumab was available for HER2-positive disease. As many of these biases have now been overcome and trastuzumab administration is standard for HER2-positive tumors, future results are expected to illustrate correlations between outcomes and molecular sub-types better.

### Assessing Tumor Molecular Sub-Type, Standard Risk Factors, and Outcome

One major issue was, and still is to a certain extent, whether outcome was linked to tumor sub-type as well as to well-known risk factors. When compared with histology, tumor size, and margin status, biological sub-types did not emerge as significant risk factors for LRR in a multivariate analysis of 1,994 patients (45% of luminal HER2 and 53% of HER2-positive received trastuzumab) (38). On the other hand, HER2 and TN subtypes appeared to be risk factors for time to LR, together with older age at diagnosis and RT dose to the whole breast in a multivariate analysis of 1,434 patients treated with BCS and WBI (43). HER2-positivity, TN and Luminal B sub-types, number of positive lymph nodes, and younger age emerged as risk factors for LR in 2,233 patients (42). Multivariate analysis showed that hormonal receptor-positive/HER2-positive, hormonal receptor-negative/HER2-negative phenotypes, and number of positive nodes were associated with shorter LRR-free survival in 819 patients who did not receive post-mastectomy RT. Age over 50 years was associated with longer LRR-free survival (36).

Despite apparent divergencies as studies did not analyze the same risk factors, these results throw light on the difficulties in achieving definitive evidence of the impact of molecular sub-type upon outcomes.

### Assessing Type of Surgery, Tumor Molecular Sub-Type, Post-Operative RT, and Outcome

Current evidence suggests type of surgery should not vary with tumor molecular subtype in an attempt to improve outcomes. A systematic review of 15 studies enrolled 12,592 patients. After BCS and post-operative RT in 7,176 patients, luminal tumors were linked to a lower risk of LRR than HER2-positive and TN tumors; the risk was higher in HER2-positive than in TN tumors. After mastectomy in 5,416 patients, followed by RT in 44%, luminal tumors had a lower risk of LRR than HER2-positive and TN tumors, both of which had similar risks. In five of these 15 studies with comparable data for patients who underwent mastectomy or BCS followed by RT, LRR was independent of surgery in TN tumors and was lower after mastectomy in luminal and HER2-positive subtypes (44). In another meta-analysis 8/22 studies compared recurrence rates after BCS and mastectomy in patients with TN tumors, showing the LRR and DM rates were significantly lower after BCS (30). Biases such as retrospective studies, different disease stages and follow-up times, old and/or unspecified schemes of adjuvant systemic therapies, trastuzumab administration to very few patients, and few events in some series, precluded drawing conclusions on the best surgical approach according to sub-type.

Although the next challenge was to determine whether post-operative RT impacted upon outcomes, reports of its benefits were divergent because no study was designed to link post-operative RT, outcomes, and different sub-types. In a retrospective analysis of 2,118 primary operable breast cancer with diverse subtypes, post-operative RT impacted significantly on relapse-free survival only in the Luminal A sub-type (35). BCS + RT were associated with a significantly lower risk of LRR than mastectomy alone in T1-2N0 TN breast cancer patients but post-mastectomy RT nullified this difference (45).

Other studies investigated whether tumor sub-type was predictive of RT benefit after mastectomy. In trials 82 b and c, the Danish Breast Cancer Cooperative Group (DBCG) randomized 3,083 high-risk breast cancer patients to post-mastectomy RT or not. Bio-pathological features were analyzed in 1,000 by staining tissue microarray sections for ER, PR, and HER2. At a median follow-up of 17 years for surviving patients, post-mastectomy RT significantly reduced the probability of LRR in receptor-positive and HER2-negative tumors, receptor-negative and HER2-positive tumors and TN tumors but was associated with significantly better OS only when tumors were hormonal receptor positive and HER2-negative (46). In a merged analysis of the British Columbia and DBCCG 82b trials on premenopausal patients, post-mastectomy RT significantly lowered LRR in Luminal A tumors and, to a lesser extent, in basal-like tumors. The small cohort may account for the lack of significance in the other sub-types (47). In a US national comprehensive cancer network report, post-mastectomy RT was administered to 30% of 5,673 patients with stage I-III breast carcinoma. Its effect on LRR was greater in Luminal A than B while it had no significant effect on TN patients or in the HER2- positive group who did not receive trastuzumab (37).

After BCS a 6- immunohistochemistry-marker subtyping panel analyzed tissue samples from 501/769 node negative patients. They were enrolled in the Toronto-British Columbia randomized clinical trial to receive tamoxifen or tamoxifen plus RT. RT significantly reduced the cumulative incidence of LRR in high-risk sub-types but not in Luminal A and B tumors due to, perhaps, the few relapses in these subgroups. Although patients with luminal tumors benefitted less from RT than other sub-types, the interaction between RT and sub-type was not significant (40). Different results for 958 tumors emerged from the Swedish Breast Cancer Group 9 Radiotherapy (SweBCG91-RT) trial which used immunohistochemistry and *in situ* hybridization of tissue microarrays. One thousand three patients with node-negative, stage I and II breast cancer were randomly assigned to BCS with or without RT; only 8% received systemic adjuvant therapy. RT significantly reduced the cumulative incidence of LR as a first event within 10 years for Luminal A and B–like tumors. No significant effect was seen on HER2-positive or TN tumors, the latter perhaps because very few patients had this sub-type. Death from any cause was not improved by RT in any sub-type but breast cancer-related mortality was reduced in TN tumors (48).

Overall, RT significantly reduced the risk of LRR in mastectomized Luminal A patients, but its impact was less clearly defined after BCS (35, 40, 46–48). Disease stage may account for these divergencies, as mastectomized patients had high-risk lymph node positive disease (46, 47) while BCS patients had T1-2N0 disease (40, 48). Differences in cohort size, number of events, and administration of adjuvant systemic therapy may also have played roles in BCS outcomes.

## Genomic-Driven Personalized in Adjuvant Radiation Therapy

Genomic analysis appears to be a potentially powerful tool to improve risk stratification and personalize approaches to RT, as individual gene profiles may overcome the limitations of bio-pathological markers of molecular sub-types and might succeed where other approaches have not. Molecular signatures may, however, be unable to account for the complexity of the radiation response due to the heterogeneous biology of breast cancer. Furthermore, translating laboratory-derived molecular signatures into standardized, clinically available tests is a complex task.

### Assessing Genomic Profiling/Classifiers, Risk Stratification, and Radiosensitivity

DNA microarray analysis of the primary tumor was performed in 94 patients who underwent mastectomy without RT, some of whom developed LRR after a minimum 3-year follow-up. Two distinct gene expression profiles with, respectively, 258 and 34 genes, emerged as significant predictors of LRR. Multivariate analysis revealed that besides ER status, the genomic predictive index was the only other independent prognostic factor of LRR and might potentially be used to select patients for post-mastectomy RT (49).

To identify genes which could predict whether post-mastectomy RT would reduce LRR, frozen tumor tissue specimens were analyzed from 191 high-risk mastectomized patients who were randomized to RT or not. Gene-expression analysis identified seven genes and a weighted gene-expression index (DBCG-RT profile) was able to separate patients into high and low LRR risk groups. It might identify patients who are most likely to benefit from post-mastectomy RT as it impacted significantly on the risk of LRR only in high-risk patients (50).

In gene expression profiling, the wound-response signature, 70-gene prognosis profile and a hypoxia-induced profile had been shown to predict metastasis-free survival and OS. They were investigated as LR predictors in 295 patients who received BCS followed by whole breast irradiation (WBI). Only the 512 gene “wound” signature distinguished low- from high-risk patients (51). Hierarchical cluster analysis found the two main clusters were not linked to LR in 165 primary invasive breast cancers who were treated with BCS followed by WBI, 56 of whom (34%) were relapsing premenopausal patients with pT1 or pT2 disease. Although molecular sub-types and chromosomal instability signatures were associated with LR (52) they were not validated in a larger, independent data set (53).

ther approaches aimed at correlating genomic predictors of radiosensitivity with outcome. A radiosensitivity index (RSI) that had been clinically validated in 3 independent datasets of different tumors (54–56) was tested in 159 breast cancer patients from the Karolinska University Hospital and 344 from the Erasmus Medical Center. In both datasets the RSI correlated with the risk of DM, suggesting it might serve as a predictive tool for RT efficacy (57). When RSI was combined with molecular sub-types, it distinguished two subgroups in TN patients. One bore radioresistant tumors and was at increased risk of LR while the other displayed similar radiosensitivity to luminal patients. In multivariate analysis radiosensitivity combined with molecular sub-type and age emerged as the most significant predictors for LRR (58). In an attempt to develop radiosensitivity signatures intrinsic radiosensitivity ranged from 17 to 77% in 16 breast cancer cell lines (5 luminal, 4 basal A, 4 basal B, 3 HER2/*neu* amplified) which were tested in radiation clonogenic assays (RSS). They were associated with 147 genes (80 negatively; 67 positively) even though they did not correlate significantly with tumor sub-types. A 51-gene RSS which was elicited in a training cohort of patients who had been treated with post-operative RT, was validated in an independent series of 228 cases, most of whom had received RT. At 10 years, the RSS predicted the risk of LRR with sensitivity and negative predictive values of 84 and 89%, respectively, outperforming clinical factors (59).

To predict the benefit of RT, gene expression signatures were developed on the basis of intrinsic radiosensitivity in 948 patients and of anti-tumor immunity in 129. Since radiosensitivity was significantly associated with loco-relapse free survival, the signature was validated in a cohort of 1,439 patients and a trend towards benefit was observed in the radiation-sensitive *vs* the non-radiation sensitive. RT did not impact on disease-specific survival which, however, was significantly better in the immune-effective group. Integrating the two signatures predicted RT benefit better. Validation in a prospective randomized trial is, however, needed before the radiosensitivity or anti-tumor immunity signatures might eventually be adopted in clinical practice (60).

Another approach to personalizing RT is the genomic-adjusted radiation dose (GARD). Derived from combining the gene-expression-based radiosensitivity index (54–56, 61) with the linear quadratic model, GARD emerged as the only independent predictor of DM-free survival in 263 patients with clinical T1-T3N0 breast cancer who underwent BCS and WBI. GARD was significantly associated with relapse-free survival in a cohort of T1-T3, N0-N1 patients (61). Hypothesizing that GARD could predict LR, it was tested in two independent datasets of patients with TN tumors. The first enrolled 58 patients treated with BCS and post-operative RT to the breast plus/minus draining nodes while the second included 55 patients who received BCS or mastectomy, followed by post-operative RT. Since GARD was significantly associated with local control in both, a model was developed to tailor the RT dose to each patient. It showed that doses up to 70 Gy may be needed for some patients despite the increased risk of toxicity (62).

To tailor response to RT the Adjuvant Radiotherapy Intensification Classifier (ARTIC) was developed from three datasets of early-stage breast cancer patients who were treated with RT. Comprising 27 genes and the patient’s age, data included details of gene-expression and LR. In its validation for LRR in 748 patients, ARTIC emerged as a highly prognostic tool in patients treated with RT. When ARTIC scores were low, RT significantly reduced the 10-year cumulative incidence of LRR; high ARTIC scores were associated with less benefit from RT. As 88% of LR occurred in the same quadrant as the primary tumor in the high-risk group and 85% of regional relapses in the axilla, some patients would have benefitted from intensified RT schedules such as tumor-bed boost and regional nodal RT (63). ARTIC should be re-validated in patients treated with modern systemic adjuvant strategies since the high relapse rate may have been due to adjuvant systemic therapy being administered to a low percentage of patients.

Even though some molecular signatures/classifiers have been developed to predict DM, LRR rate, and/or tumor response to radiation, none is, as yet, approved for clinical use mainly because the gene profiles differed greatly and impacted outcomes differently. Clinical validation of gene signatures is arduous due to lack of standardization in RNA extraction and differences in patient and treatment selection. Results were derived from retrospective, often small, cohorts with diverse RT doses and volumes and, when reported, systemic therapy schedules were generally old. Furthermore, routine gene profiling for individual patients is far too expensive for clinical practice (64, 65).

**Table 2** (66–69) reports other studies on this topic.

**Table 2:** Gene expression and outcomes in breast cancer patients.

Author	Analysis	Major Results
Niméus-Malmström et al. (66)	Gene expression analysis on RNA in 143 patients	**LR** ER+ *vs* ER− ROC areas (0.91, p = 9 × 10^−6^ *vs* 0.74 p = 0.08)
Le Scodan et al. (67)	A quantitative reverse transcriptase PCR-based approach measured mRNA levels of 20 genes in 97 patients	**RAD51 was the only gene associated with:** **5-yr LRR-free survival** 100% (low RAD51) *vs* 70% (high RAD51), p < 0.0001 **5-yr OS** 95% (low RAD51) *vs* 69% (high RAD51), p = 0.0002
Meng et al. (68)	Gene expression microarrays analysis	**IDC DFS** Was related with MAMDC2, TSHZ2, and CLDN11, p < 0.001 **OS** Was shorter with high CLDN11 expression, p = 0.012
Jang et al. (69)	Transcriptional and mutational profile analysis by scRNA-seq	**RR cells in basal subtype were related to:** high PD-L1, p < 0.001 high TMB, p = 0.033

LR, local relapse; ROC areas, areas under the receiver operating curve; LRR, loco-regional relapse; OS, overall survival; IDC, invasive ductal carcinoma; DFS, disease-free survival; RR, radioresistance; TMB, tumor mutation burden.

### Assessing Genomic Tests and Outcome

Following in the footsteps that guide clinicians in the choice of adjuvant systemic therapy, studies attempted to stratify patients by means of commercially available small gene sets. To identify suitable breast cancer candidates for adjuvant RT, genomic tests investigated risk subgroups and LRR and whether the relationship varied with the type of local treatment.

The Oncotype DX 21-gene RS significantly associated RS with LRR risk in node-negative, ER-positive patients from the National Surgical Adjuvant Breast and Bowel Project (NSABP) B-14 and B-20 trials, who had received BCS and WBI or mastectomy, followed by tamoxifen (895 patients), placebo (355), or chemotherapy plus tamoxifen (424). In multivariate analysis, RS emerged as a significant independent predictor along with age and type of initial treatment, suggesting it might discriminate between candidates for post-operative RT (70).

RS was not associated with LRR in 110 ER-positive patients who received BCS followed by RT. On the other hand, in 53 mastectomized patients it seemed helpful in selection for post-mastectomy RT as, at a median follow-up of 68.2 months, an RS > 24 predicted a higher LRR rate (71). Another series of 1,758 patients with stage I-II, ER-positive breast cancer (81% with RS ≥ 25), who had been treated with mastectomy or BCS ± post-operative RT, were retrieved from the US National Prospective Breast Cancer-Collaborative Outcomes Research Database. At a median follow-up of 29 months, risk of isolated LRR (iLRR) was not significantly associated with an RS ≥ 25 in the entire cohort. It was, however, significantly associated with an RS ≥ 25 in 74/1,199 women who had received adjuvant ET but not chemotherapy. Overall, in these 1,199 patients, higher RS was associated with greater risk of iLRR (72).

RS might be combined with standard clinical-pathological risk factors to improve LRR risk stratification and identify suitable candidates for adjuvant RT after BCS. To test this hypothesis, 388 patients were retrieved from the Eastern Cooperative Oncology Group’s database of the E2197 prospective randomized clinical trial. All had one to three positive lymph nodes or tumors >1.0 cm in size and negative lymph nodes with about 44% being receptor-negative. Neither biological subtype nor 21-gene RS was associated with LR or LRR in univariate or multivariate analyses but when analyzed as a continuous variable, the 21-gene RS emerged as a significant risk factor for LRR (73).

Other studies confirmed these findings. In 1,065 node-positive, ER-positive patients who received adjuvant chemotherapy and ET, no post-mastectomy RT was delivered and only the breast was irradiated after BCS. RS emerged as a significant predictor of LRR; multivariate analysis showed nodal status and tumor size were also independent predictors of LRR (74). In 2,326 node-negative, ER-positive/HER2-negative patients univariate analysis showed that RS category, T stage and lymphovascular invasion impacted on LRR risk. Even after adjusting for lymphovascular invasion and T stage, RS remained significantly associated with LRR. Compared with low RS, LRR risk increased 3-fold in the intermediate risk category and over 4-fold in the high-risk category (75).

RS was linked with randomized treatment, number of positive nodes and surgical type in a cohort of 316 post-menopausal, ER/PR-positive, node-positive patients who were retrospectively extracted from the Southwest Oncology Group S8814 phase 3 trial. After BCS and WBI, patients were randomized to tamoxifen alone, chemotherapy followed by tamoxifen, or concurrent tamoxifen and chemotherapy. The 10-year cumulative incidence of LRR was significantly different in each RS category (9.7% for a low RS, *vs* 16.5% for intermediate or high RS). The same profile was observed after mastectomy without RT. When patients had one to three involved nodes, a low RS was associated a 1.5% LRR rate and an intermediate or high RS with 11.1% LRR. Multivariate analysis confirmed that a higher RS was a predictor of LRR (76).

Over time, Oncotype DX has used different RS definitions for systemic therapy. The original cut-offs were <18, 18–30, and ≥31 but more recently, the TAILORx trial set cut-offs at <11, 11–25, and ≥26 in order to minimize the risk of systemic therapy under-treatment in potentially high-risk patients (77). A discrepancy in use of different cut-offs in the 21 gene RS is worth nothing. Although all ongoing RT trials and most research selected the original <18 cutoff to identify low-risk patients when aimed at defining a role for post-operative RT, in clinical decision-making for systemic therapy the <11 threshold is now used. An open question is whether the same consensus on RS cut-offs is advisable for systemic therapy and RT.

The EndoPredict test did not appear to be useful in tailoring local therapy in patients at low-risk of LRR. In 1,324 postmenopausal patients who were selected from a cohort of 3,714 that had been randomized to receive tamoxifen or tamoxifen followed by anastrozole, it classified 683 at high risk and 641 at low risk of recurrence. At a median follow-up of 72.3 months, the risk of LR was significantly higher in high-risk than in low-risk patients. LR rates were similar after BCS and mastectomy. After BCS, RT significantly improved LR-free survival in both low- and high-risk sub-groups (78). The predictive role of PAM50 on LR was assessed in 1,308 HER2-negative patients from the same trial. The risk of recurrence (ROR) score was an independent predictor of LR-free survival independently of nodal status, tumor size, and patient’s age. The 10-year LR-free survival was significantly lower in patients with a ROR score of ≥57 (79).

The 70-gene signature (MammaPrint™) emerged as an independent prognostic factor for LRR. The LR risk was significantly lower in 561 low-signature T1-3N0-1 patients who were treated with BCS and RT or mastectomy at the Netherlands Cancer Institute, than in 492 with a high signature. The 70-gene signature emerged as a prognostic factor for LRR in a competing risk analysis which included clinical-pathological risk factors such as age, tumor size, grade, hormone receptor status, lymphovascular invasion, axillary lymph node involvement, surgical treatment, ET, and chemotherapy (80).

Finally, studies investigated whether the Oncotype DX assay and RT impacted upon OS. An observational cohort study enrolled T1-2N1 ER-positive patients, some of whom received post-mastectomy RT. The National Cancer Database (NCDB) provided 7,332 patients and the Surveillance, Epidemiology, and End Results (SEER) registry supplied the validation cohort of 3,087 patients. In both cohorts RS and post-mastectomy RT interacted significantly with OS but post-mastectomy RT was associated with longer OS only when RS was low. Thus caution should be exercised when omitting post-mastectomy RT in women with low RS (81). In a pooled analysis of 1,778 patients from seven clinical trials, all had stage I, ER- and/or PR-positive, HER2-negative disease, and an Oncotype RS no greater than 18. After BCS ± post-operative RT they had received ET but not chemotherapy. The 5-year relapse-free interval was significantly lower in the post-operative RT group. RT omission significantly increased the risk of LRR, but not of DM, breast cancer-specific survival or OS. The RT effects varied across subgroups, with lower relapse-free interval rates in older patients with RS under 11 (*vs* 11–18) and ER-positive/PR-positive status (*vs* other) (82). Other studies on this topic are reported in **Table 3** (83–85).

**Table 3:** Studies assessing genomic tests and outcome.

Author	N° patients	Treatment	Follow-up	Results
Dong et al. (83)	13,246	BCS ± RT	NA	**Postoperative RT: independent predictor of better BCSS only in intermediate risk (RS) group** (HR 0.630; 95% CI 0.416–0.955, p = 0.029)
Wu et al. (84)	18,456	BCS ± RT	NA	**Postoperative RT: independent predictor of better BCSS only in intermediate risk (RS) group** (HR 0.467; 95% CI 0.283–0.772, p = 0.003)
Zhang et al. (85)	1,571	Mastectomy ± RT	30 months	**5-y BCSS in the high risk group** No PMRT subgroup 100.0% *vs* PMRT subgroup 90.0%, (p = 0.046) **No significant difference in BCSS in the PMRT group *vs* the no PMRT group (p = 0.427)**

BCS, breast conserving surgery; RT, radiotherapy; BCSS, breast cancer-specific survival; RS, recurrence score; PMRT, post-mastectomy radiotherapy.

To help fill current gaps between adjuvant systemic therapy and RT in clinical practice and individualize prediction of RT outcomes, larger validation studies are warranted to define genomic predictors and their values in improving health care.

## Discussion

Personalized medicine in radiation oncology for breast cancer aims at improving survival outcomes and quality of life as well as reducing treatment-related morbidity and National Health Service costs. Reaching this goal is arduous because so many factors impact upon outcomes. In order to throw some light on the topic, the present overview explored the links between adjuvant RT, type of surgery, and the response of each sub-type to RT, finding study limitations precluded definitive conclusions. The earliest studies investigated whether diverse molecular sub-types impacted on LR and/or LRR, which is the most common RT-related outcome and a well-established predictor of DM, mortality and survival (86–88). Attention also focused on whether sub-type and type of surgery (BCS or mastectomy) were predictive of outcome but no firm evidence emerged to support one type of surgery over another, so choice of surgery remains dependent on standard criteria, such as breast dimension and/or tumor extension and patient’s choice.

After finding Luminal A tumors were associated with a low risk of LRR they emerged as highly radiosensitive. HER2-positive tumors were associated a high risk of LRR and radioresistance which was reversed by trastuzumab administration (37, 89–93). Finally, drugs could not overcome the high risk of LRR and radioresistance in the TN subtype as there were no effective treatment targets. On the other hand, RT was reported to lower the risk (45, 46, 90, 91), even though the benefit was less evident than in the luminal and hormone-receptor positive subtypes. Post-operative RT also seemed to account for a lower relapse rate after BCS than after mastectomy (92).

Gene expression profiling appeared to offer a pathway to tailored RT and when small gene sets were evaluated as predictors of LRR or OS risk, results appeared promising. Despite some interesting results no signature has, however, as yet been approved or validated for clinical use. To ensure that tailored RT for breast cancer becomes a clinical reality, present efforts, in our view, should be directed towards validation studies that focus on the most promising biomarkers as they are crucial in identifying appropriate patients for RT escalation or de-escalation schedules. Nowadays, ongoing RT de-escalation trials that are based on biomarkers and genomic profiling (77, 93–99) seek to better stratify the LR risk and identify patients who can omit RT after BCS. Moreover, one ongoing trial was designed to assess whether RT was needed after mastectomy and whether treatment volumes should be adjusted in patients with pT1-2N1a who are ER-positive, HER2-negative and at low biological risk (21-gene RS < 18) (100, 101). The results are expected to provide future recommendations for personalized RT.

Predictive biomarkers may perhaps be validated by exploiting information from large databases (102) which may combine the anatomic extent of disease with biological factors like grading, ER, PR, and HER2 status. These were in fact included in the 8^th^ Edition of the AJCC staging manual (103). Once suitable genetic assays are validated for adjuvant RT, their use will be easily incorporated into clinical practice as such kits are already used to identify suitable patients for adjuvant chemotherapy and are more accurate than clinical-pathological features.

The present overview has illustrated the potentialities of molecular sub-types and genomic profiling but also uncertain results and lack of definitive conclusions. To overcome today’s lack of over-arching strategy, research groups are advised to collaborate on a shared approach, bearing in mind that achieving personalized radiation oncology in breast cancer will require specific infra-structure, networking and investment (104). Besides focusing on clinical biomarkers, molecular signatures, tumor phenotypes, and genomics, research will also need to incorporate RT technical aspects, imaging, radiomics as well as patient-related factors like genetics and genetic predisposition, comorbidities, lifestyle, and environmental features. Even data on breast tissue composition and its microenvironment may contribute to personalizing the approach to the patient.

## Author Contributions

CA conceptualized and designed the study. EA, FM, and VM acquired the data. CA and EP analyzed and interpreted the data. CA and EA drafted the manuscript. GI and SS critically revised the manuscript. All authors contributed to the article and approved the submitted version.

## Conflict of Interest

The authors declare that the research was conducted in the absence of any commercial or financial relationships that could be construed as a potential conflict of interest.
